# Monitoring and treating fetuses with gastroschisis using the Svetliza Reducibility Index (SRI) and the EXIT-like procedure - a novel approach

**DOI:** 10.1590/S1679-45082017ED4238

**Published:** 2017

**Affiliations:** 1 Disciplina de Cirurgia Pediátrica e Transplante Hepático Pediátrico, Faculdade de Medicina, Universidade de São Paulo, São Paulo, SP, Brazil.

By and large, Medicine and sciences only advance due to new ideas, which should be tested according to academic standards to be accepted. It is estimated that out of every 100 new and creative ideas, few will effectively give some contribution and lead to improvement. However, remembering Thomas Alva Edison “There's a way to do it better - find it!”^(^ ^1^ ^)^ Therefore, new ideas should not be despised, *a priori,* as long as they are logic and consistent.

The article under analysis^(^ ^2^ ^)^ is based on and is a recombination of several ideas that appeared over the last 20 years, which are still evolving and are far from their finalization. Gastroschisis, due to the difficulties in treatment and tumultuous progression, is a malformation favorable as testing ground of new ideas.

The ideal treatment consists of early and complete reduction of all the exteriorized bowel loops in the least traumatic manner possible, which is not always attainable.

Bedside reduction of the herniated loops to inside the abdominal cavity with no anesthesia is known and applied since 1998, as described by Bianchi et al.^(^ ^3^ ^)^ The advantages of immediate or early reduction of the loops are also frequently mentioned by other authors, including Kimble et al., 2001.^(^ ^4^ ^)^

Whereas an elective early delivery in order to carry out an easier reduction of the loops, hypothetically less damaged and swollen due to their exposure to amniotic fluid, although mentioned,^(^ ^5^ ^)^ is far from being generally accepted.^(^ ^6^ ^)^ It has been sufficiently demonstrated by full-term babies born presenting with sparse serositis, as well as premature infants born with intense edema and bowel loop congestion.

Quantification of progression of edema in the protruding loops by ultrasound to anticipate birth of a child with good viability, is an interesting measure to be discussed. However, the cited Svetliza Reducibility Index (SRI), which uses the diameter of the largest dilated loop, thickness of this loop wall, and assessment of the abdominal wall ring, is not a part of the original paper by Svetliza et al.^(^ ^7^ ^)^ Although the reduced diameter of the abdominal wall ring interferes in the difficulty to reduce the loops, and probably is also correlated with the thickness of the loop wall due to venous congestion, *per se,* it is not a deterrent factor for their reduction. However, surgical widening is necessary, which would not be compatible with the reduction without anesthesia, but could provide a solution to the problem.

The use of ex-utero intrapartum treatment (EXIT) to treat the malformation, especially in cases of diaphragmatic hernia, was developed by Michel Harrison, in San Francisco, in 1997.^(^ ^8^ ^)^ Its use in treatment of gastroschisis was initially described by Zhang et al.,^(^ ^9^ ^)^ in 2010, in two cases accompanied by a discussion about anesthesia in this type of procedure, by Luo et al.,^(^ ^10^ ^)^ also in China, in 2015.

The recent participation of Dr. Javier Svetliza in conferences and the publication of his experience in 2011,^(^ ^7^ ^)^ associated to Kimble et al.,^(^ ^4^ ^)^ early reduction and no anesthesia, according to Bianchi et al.,^(^ ^3^ ^)^ using the placental circulation of Harrison^(^ ^8^ ^)^ EXIT along with Zhang et al.,^(^ ^9^ ^)^ procedure, raised a lot of interest due to the possibility of improving the final results of gastroschisis. This justified the preparation of the project herein analyzed.

Reporting some new and instigating concepts is commendable, but these should be confirmed by a greater numbers of cases, as mentioned by the authors. Nevertheless, to show better results regarding time until full feeding, need for mechanical ventilation, time until hospital discharge, and mortality, we should compare the results of EXIT-like reduction with pure and simple early reduction, performed in the operating room, just beside the delivery room, with or without anesthesia, and not compare the cases that used improvised PVC surgical silos, which knowingly result in a worse prognosis and clinical progression.

It is difficult to believe that a mere five-minute difference between treating the patient on the mother's legs, still linked to the uterus, or in the operating room next door could make such a difference in the end. However, only randomized series (perhaps multicenter studies) can confirm the propositions by Svetliza as superior to those traditionally used.
